# Cause and Treatment of Prolapsus of the Rectum

**Published:** 1854-07

**Authors:** M. Duchassay


*Cause and Treatment of Prolapsus of the Rectum.* By M. Duchassay. (Archives Generales de Med., Sept.)

In a short but interesting memoir, M. DUCHAUSSAY reviews the circumstances attending this troublesome complaint and fixes attention in particular upon the loss of power in the sphincter and muscle as the chief cause of the descent of the bowel. Moreover, he endeavours to show that Dupuytren's operation, by excising the radiating folds of skin around the anus, and the operation by four touches with the actual cautery, practiced by Guersant, act not by causing any subsequent retraction of the cellular tissue, skin, and mucous membrane, but rather by stimulating the sphincter muscle so that it regains its contractility, and therefore its retentive character. How else, asks M. Duchaussay, do we explain the fact, that the prolapsus is often cured, or does it return after two days, or even after one day, or not at all after the operation? He points out the fact, that in cases of this disease in infants, three fingers may sometimes be introduced without causing contraction of the sphincter, before the operation by cautery, whilst afterwards, if one be passed, a powerful contraction of the sphincter immediately ensues. As proof that this recovery of contractile power by the sphincter is the cause of cure, a case is mentioned in which M. Guersant had used the cautery too superficially, the sphincter failed to contract, and the disease returned. A second cauterization was followed, on the contrary, by return of the muscular contractility, and the cure was complete.

According to the author, the cautery acts as a stimulant to the paralyzed muscle, just as it will to the deltoid in a like condition. After pointing out the inconveniences and apparent severity of M. Guersant's method, M. Duchaussay suggests that a slighter cautery, or some other stimulant to muscular contractility, might act as well, and he suggests strychnine. This, with M. Guersant's permission, has been tried in the Hopital des Enfants, in the case of a girl aged eleven years. The prolapsus here arise from obstinate constipation; it had lasted for four years; the bowel protruded at each evacuation about ten centimeters (—4 inches).—During the first month of her admission she was treated by laxatives only, with no other result than that of diminishing the length of the protruded portion of bowel to about four centimeters ( $1\frac{1}{2}$ inches.) Strychnia was then employed endermically near the region of the sphincter: the next day there was no evacuation; on the following day the bowels acted once, only a slight bulging of the rectum taking place; on the third day the protrusion was still less after an ordinary evacuation; and during the next thirteen days it did not occur again.

Blisters were made in the cleft between the nates, and on the right thigh close to that cleft; one sixth of a grain of strychnia was applied the first day, one third on the second, and one third on the fourth day. On the fifth day, about half a grain of sulphate of strychnia was used, and this was repeated for the last time on the sixth day. In the case of a boy, it is recommended to be applied between the scrotum and anus, immediately over the anterior interlacement of the sphincter ani fibres. The remedy certainly deserves further trial.

